# Editorial: Immunological aspects of emerging and re-emerging zoonoses

**DOI:** 10.3389/fimmu.2024.1392382

**Published:** 2024-03-06

**Authors:** Wei Wang, Jingxin Li, Yuejin Liang, Wenping Gong

**Affiliations:** ^1^ National Health Commission Key Laboratory on Parasitic Disease Prevention and Control, Jiangsu Provincial Key Laboratory on Parasites and Vector Control Technology, Jiangsu Institute of Parasitic Diseases, Wuxi, Jiangsu, China; ^2^ Jiangsu Provincial Medical Innovation Center, National Health Commission Key Laboratory of Enteric Pathogenic Microbiology, Jiangsu Provincial Center for Disease Control and Prevention, Nanjing, Jiangsu, China; ^3^ Department of Microbiology and Immunology, University of Texas Medical Branch, Galveston, TX, United States; ^4^ Senior Department of Tuberculosis, the Eighth Medical Center of Chinese People’s Liberation Army (PLA) General Hospital, Beijing, China

**Keywords:** zoonoses, viral disease, immune response, immunodiagnostics, immunotherapeutics, COVID - 19

Zoonoses, a group of diseases that are transmitted between animals and humans, are considered as the most prevalent infections in humans (1). They present substantial global health threats and continue to pose significant challenges to both the scientific community and public health systems (2). Currently, approximately 60% of identified infectious diseases and 75% of emerging infectious diseases originate from zoonotic sources (3). Globally, zoonoses are estimated to be responsible for 2.5 billion cases of human illness and 2.7 million human deaths annually, with an additional 5 to 6 million individuals at risk of contracting zoonotic infections (4). Emerging and re-emerging zoonoses are therefore considered as major and global challenges for public health in terms of their profound health impacts and economic burdens (5). Recently, the One Health approach has been widely recommended to address the challenges of zoonoses (6, 7).

The current Research Topic consists of 9 publications including 6 original research articles and 3 review articles, covering immune responses, immunodiagnostics and immunotherapeutics of zoonoses, with the goal of improving the understanding of the immunological facets of emerging and re-emerging viral zoonoses in this changing world. This editorial aims to provide an overview of the contributing articles within this Research Topic and highlight their significance in advancing our understanding of zoonotic diseases.

Coronaviruses are a large family of RNA viruses that may infect both humans and animals (8). Coronavirus disease 2019 (COVID-19), caused by the coronavirus severe acute respiratory syndrome coronavirus 2 (SARS-CoV-2) (9), has been recognized as one of the deadliest infectious diseases in human history responsible for millions of human fatalities and trillions of economic losses (10, 11). A large number of studies have been therefore conducted with the aim to provide insights into the containment of the COVID-19 pandemic worldwide (12). In the current Research Topic, five articles focus on COVID-19. Jeon et al. found that increased interferon alpha 1 (IFNA1) and reduced interleukin (IL)-12p40 were closely linked to persistent hyperinflammation in COVID-19 pneumonia patients. This suggests that the aberrant persistence of pulmonary and systemic inflammation might be associated with long COVID-19 sequelae. Serwanga et al. detected faster and stronger anti-SARS-CoV-2 spike-directed IgG, IgM, and IgA antibody responses in Ugandan asymptomatic COVID-19 patients compared to those with mild symptoms during acute infections. The Spike IgG antibody levels, peaking between 25 and 37 days and persisting for 28 months, exhibited significantly greater and more durable than nucleoprotein and receptor-binding domain (RBD) IgG antibody levels. In addition, they found a significantly positive correlation between Spike-and RBD-directed IgG antibody levels until 14 months of SARS-CoV-2 infections, indicating significant and persistent anti-spike immunity without RBD. Yang et al. reviewed the immune response features of immunodominant epitope-specific T cells targeting different SRAS-CoV-2 proteome structures following SRAS-CoV-2 infections and COVID-19 vaccination, analyzed the heterogenous phenotypic characteristics of SARS-CoV-2 specific T cells, and discussed the significant implications of cross-reactivity of human coronaviruses, SRAS-CoV-2 and variants of concern. Guan et al. generated two Chinese national standard candidates for anti-SARS-Cov-2 neutralizing antibody traced to the WHO International Standard according to the WHO manual for the establishment of national secondary standards. These candidates facilitate the development and potential application of COVID-19 vaccines in China. Abdelaziz and colleagues generated a multi-epitope vaccine (PanCoVac) that encoded the conserved T cell epitopes from all structural proteins of coronaviruses. This vaccine was *in vitro* processed and presented by HLA-A*0201, and immunization of NILV-PanCoVac (PanCoVac cloned into a non-integrating lentivirus vector at a single-low dose following infection with ancestral SARS-CoV-2) resulted in the absence of COVID-19-like symptoms and significantly reduced SARS-CoV-2 viral loads in lung specimens in Roborovski dwarf hamsters. This protective effect was observed at early stage (2 days post infection) after challenge and was not dependent on neutralizing antibodies, suggesting that PanCoVac may protect from severe disease caused by SARS-CoV-2 variants and future pathogenic coronaviruses. The great success of COVID-19 messenger RNA (mRNA) vaccines, as evidenced by the 2023 Nobel Prize in Physiology or Medicine (13), urges the research and development of novel RNA vaccines (14). Bai et al. summarized the discovery, synthesis, biological functions and metabolism of circular RNA (cirRNA), outlined the progress of cirRNA vaccine research, and provided an overview of production process and quality control of cirRNA vaccines.

Swine acute diarrhea syndrome coronavirus (SADS-CoV), belonging to the alpha coronavirus family, is a newly discovered, highly pathogenic coronavirus associated with acute diarrhea and mass piglet deaths (15). Huang et al. found that the nonstructural protein 5 of SADS-CoV remarkably suppressed Sendai virus (SEV)-induced production of IFN-β and inflammatory cytokines TNF-α, CXCL10, RIG-I, ISG15, RSAD2, ISG56, and IFIT3. In addition, SADS-CoV nsp5 was found to target and cleave mRNA-decapping enzyme 1a (DCP1A) via its protease activity to inhibit the IRF3 and NF-κB signaling pathways in order to decrease IFN-β and inflammatory cytokine production. Moreover, a form of DCP1A with a mutation in the glutamine 343 residue showed resistance to nsp5-mediated cleavage and exhibited enhanced capability in suppressing SADS-CoV infection compared to wild-type DCP1A, indicating the significance of SADS-CoV nsp5 as an interferon antagonist.

Mpox (formerly known as monkeypox) is a zoonotic viral disease caused by the mpox virus, primarily found in central and western Africa (16). Nevertheless, a sudden outbreak of mpox occurred, rapidly spreading across Europe, the Americas, and subsequently affecting all six WHO regions since May 2022 (17). To coordinate global efforts to address this challenge, WHO declared the mpox outbreak a public health emergency of international concern on July 23, 2022 in the context of the COVID-19 pandemic (18). In this Research Topic, Niu et al. summarized the origins and transmission routes of mpox virus, as well as insights into the epidemiology, pathophysiology, clinical manifestations, diagnosis, treatment and vaccines of mpox. This synthesis offers a detailed understanding of this disease.

Tick-borne encephalitis virus (TBEV) is a neurotropic flavivirus causing tick-borne encephalitis, which is associated with severe neurological disease, long-term neurological sequelae or death (19). Langat virus (LGTV), a naturally attenuated member of the TBEV complex, has been found to be lowly virulent in humans and shares a more than 80% amino acid homology with TBEV (20). Therefore, LGTV has been attempted as a live attenuated vaccine for prevention of TBEV infection (21, 22). In this Research Topic, Kubinski et al., found that LGTV infection induced both cross-reactive antibodies and T cells against TBEV in C57BL/6JOlaHsd (BL6) mice, and sera from LGTV-infected mice efficiently protected from developing severe tick-borne encephalitis, while adoptive transfer of T cells from LGTV-infected mice failed to provide this protection; additionally, histopathology of infected mouse brain specimens revealed a possible role of microglia and T cells in inflammatory processes within the brain.

While significant progress has been made in understanding immunological aspects of emerging and re-emerging zoonoses, several challenges and future directions remain. Firstly, in the context of viral zoonoses, including emerging variants, further investigations are needed to elucidate the complex interplay between viral pathogens and the immune system. Understanding the mechanisms of immune evasion employed by these viruses may inform the development of targeted countermeasures. Secondly, the global deployment of effective vaccines remains a priority. Research efforts are recommended to continue to focus on the development of safe and efficacious vaccines against zoonotic diseases, with an emphasis on rapid vaccine development platforms that respond promptly to emerging threats. Lastly, ongoing surveillance, comprehensive public health interventions, and international collaborations are essential for early detection, containment, and prevention of future zoonotic disease outbreaks.

In summary, this Research Topic offers a significant contribution to our understanding of the immunological aspects of emerging and re-emerging zoonoses. By elucidating the intricate mechanisms underlying host-pathogen interactions, immune responses, and immunopathology, these studies may provide insights into the development of effective preventive and control interventions against emerging and re-emerging zoonoses. These publications have received 26,022 views and 7,034 downloads. We expect that the topic will help accelerate efforts toward the containment of emerging and re-emerging zoonoses.

## Author contributions

WW: Conceptualization, Funding acquisition, Resources, Writing – original draft. JL: Funding acquisition, Writing – review & editing. YL: Writing – review & editing. WG: Writing – review & editing.
